# Limits on Anti-Phase Synchronization in Oscillator Networks

**DOI:** 10.1038/s41598-020-67021-6

**Published:** 2020-06-23

**Authors:** George Vathakkattil Joseph, Vikram Pakrashi

**Affiliations:** Dynamical Systems and Risk Lab, University College Dublin, Dublin, Ireland

**Keywords:** Complex networks, Nonlinear phenomena, Applied mathematics

## Abstract

**Anti-phase synchronization is the spontaneous formation of 2 clusters of oscillators synchronized between themselves within a cluster but opposite in phase with the other cluster. Neuronal networks in human and animal brains, ecological networks, climactic networks, and lasers are all systems that exhibit anti-phase synchronization although the phenomenon is encountered less frequently than the celebrated in-phase synchronization. We show that this disparity in occurrence is due to fundamental limits on the size of networks that can sustain anti-phase synchronization. We study the influence of network structure and coupling conditions on anti-phase synchronization in networks composed of coupled Stuart-Landau oscillators. The dependence of probability of anti-phase synchronization on connectivity of the network, strength of interaction over distance, and symmetry of the network is illustrated. Regardless of favourable network conditions, we show that anti-phase synchronization is limited to small networks, typically smaller than 20 nodes**.

## Introduction

Coupled oscillators and phenomena associated with them have attracted considerable attention recently not only due to the behavioural richness they exhibit but also due to their generality in capturing the essential dynamics of multiple real-world systems (e.g. Lasers^1^, Josephson junctions^2^, neuronal networks^3^, ecological systems^4^, among others^5^). Synchronization of phase oscillators has probably been the most studied phenomenon in this burgeoning field^6–10^.

The notion of synchronization we use here implies that the phases of the oscillators become and remain identical regardless of their initial conditions - usually termed as in-phase synchronization. Some form of attractive coupling is essential for the oscillators to ‘pull’ others in the network to a common phase. The first form of synchronization as observed by Huygens in pendulum clocks^5^ however, is anti-phase synchronization, where the phases of oscillators are separated by a phase difference of $\pi$. In addition to pendulums, systems such as neuronal networks in brains^11–13^, food webs^14,15^, climate networks^16,17^ and lasers^18^ have been shown to exhibit antiphase synchronization. From the simple case of two oscillators, it can be deduced that some form of repulsive coupling would be required in order to achieve anti-phase synchronization^19^. This has been shown in real networks. E.g. changes in the coupling direction in the circadian clock neurons divides the clock oscillations into two clusters as a function of day length^20^. Cultured human epileptic cells have been shown to exhibit clustering from repulsive coupling^21^.

**Figure 1:**
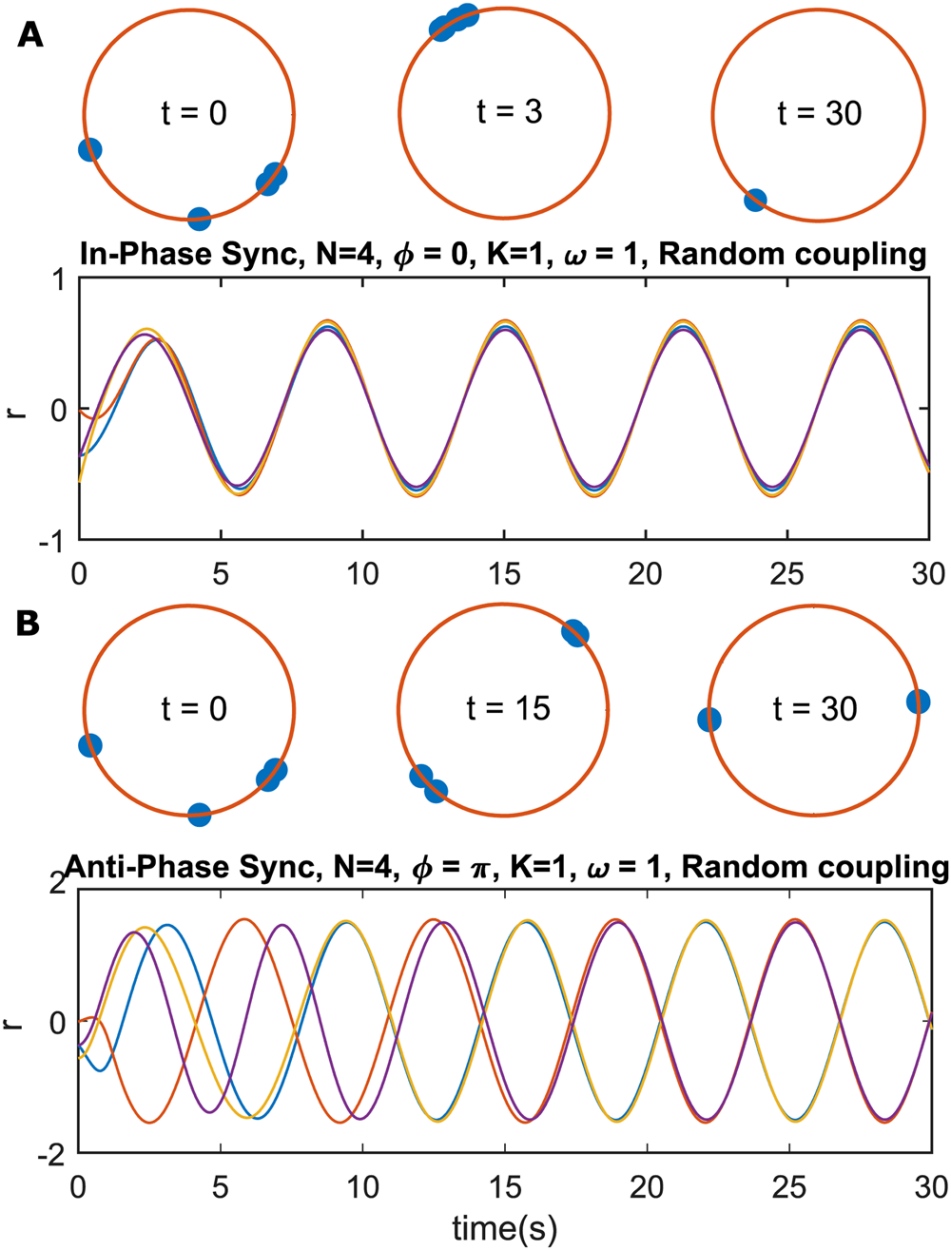
Illustration of in-phase and anti-phase synchronization. 4 identical Stuart-Landau oscillators with (**A**) purely attractive and (**B**) purely repulsive coupling, with random coupling-strengths between nodes. (**A**) exhibits in-phase synchronization and (**B**) exhibits anti-phase synchronization. ${\boldsymbol{t}}$ is time measured in seconds. The red circles show the evolution of the phase of the oscillators with time. Once locked in their respective synchronized states, both A and B remain in the same state indefinitely.

Although the two forms of synchronization may appear to be symmetric with change in coupling direction, this is not the case except for the simple 2-oscillator example. The anti-phase synchronized state becomes unstable quickly as the number of oscillators is increased. Tsimring *et al*.^22^ considered the case of identical phase oscillators (Kuramoto model), with a homogeneous global coupling and showed that for purely repulsive coupling, anti-phase synchronization *is not possible*. For non-identical oscillators, an empirical upper bound of 3 was found for the number of phase oscillators that can synchronize. In their study, the weaker phase-locking notion of synchronization was used; here we use the definition that phases should be equal (within tolerance) for in-phase (IP) synchronization and separated by 180° for antiphase (AP) synchronization (Fig. 1). Kuramoto’s phase oscillator model is a versatile approximation and often serves as a popular model for studying synchronization. However, many natural systems exhibit self-stabilizing amplitude dynamics as well, which motivated the study of the Stuart-Landau (SL) model. The SL oscillator is the simplest nonlinear extension of the harmonic oscillator and can also be viewed as the discrete form of the complex Ginzburg Landau equation^23^. This makes the SL model, near the bifurcation point of a periodic limit cycle (Hopf bifurcation), arguably the most widely applicable coupled oscillator model^24,25^.

Allowing for amplitude dynamics using the SL model allows AP synchronization of identical oscillators with purely repulsive coupling and relaxes the observed cap on oscillator count as expected (N = 4 case shown in Fig. 1). But it is reasonable to hypothesize the existence of limits on network size even for amplitude oscillators owing to the disparity in occurrence. We find that the AP synchronized solution is stable for network sizes much larger than $N=4$ in SL oscillator networks but decreases rapidly with network size beyond $N=30$ under most topologies. The effect of different features of the network topology are analysed independently and a probabilistic upper bound on the size of a network in which nodes can AP synchronize is established. The generality of the SL model allows extension of these results to real networks with amplitude dynamics and weak nonlinearity.

## Analysis

To investigate how AP synchronization is constrained, we first look at idealized conditions of network connectivity suited for the formation of two opposing clusters and progressively move towards realistic networks. We control for the following features of the oscillator network:

1. Randomness of the graph connectivity
2. Symmetry of structure
3. Distance of interaction
4. Presence of both attractive and repulsive coupling
5. Presence of non-identical oscillators

To quantify the global stability of the AP solution as the dimensionality of the system increases, the notion of basin stability introduced by Menck *et al*.^26^ is used. The method employs the volume of the basin of attraction of the desired state as measure of stability. Simply put, we count the number of random initial conditions $m$ that lead to AP synchronization and the total number of trials $N$, then $M/N$ gives us the probability of AP synchronization. Basin-stability is preferred in this case over linear stability because it is better suited for global stability analysis, can be computed easily, and is inherently normalized against an increase in dimension of the system.

An SL oscillator network can be described by a set of $N$ coupled equations in ${z}_{i}(t)$ - the complex phasor of the ${i}$ ^th^ oscillator varying with time $t$ ($t$ is dropped for notational simplicity):

1 $${\dot{z}}_{i}=(\lambda +{\mathbb{I}}\omega -{|{z}_{i}|}^{2}){z}_{i}+\frac{K}{N}{e}^{i\phi }\mathop{\sum }\limits_{j=1}^{N}{A}_{ij}[{z}_{j}-{z}_{i}]$$

where ${\rm{\lambda }}$ determines the Hopf bifurcation (a limit cycle appears at ${\rm{\lambda }}=0$), ${{\rm{\omega }}}_{i}$ is the natural frequency, $k$ is the coupling constant, $N$ is the number of oscillators and ${\rm{\phi }}$ is the coupling phase. The coupling between oscillators is given by the coupling (adjacency) matrix $A$, where each element *A*_*i,j, i≠j*_ ∈ [0, 1], *A*_*ii*_ = 0. We use $({\rm{\lambda }},K)=(\mathrm{1,3})$.

To achieve AP synchronization, from a qualitative perspective, the network should be able to self-organize into two groups as widely separated as possible from the other group, but with oscillators within the group as close as possible to each other. This makes repulsive coupling a prerequisite and we set the coupling phase ${\rm{\phi }}={\rm{\pi }}$, i.e. purely repulsive coupling. (Simultaneous presence of attractive and repulsive coupling can also lead to AP synchronization and has similar limits on network size (see later section), but elsewhere we limit ourselves to purely repulsive coupling, defined by setting the range of ${A}_{{ij}}\in [\mathrm{0,1}]$).

## Results

### Random graphs

**Figure 2:**
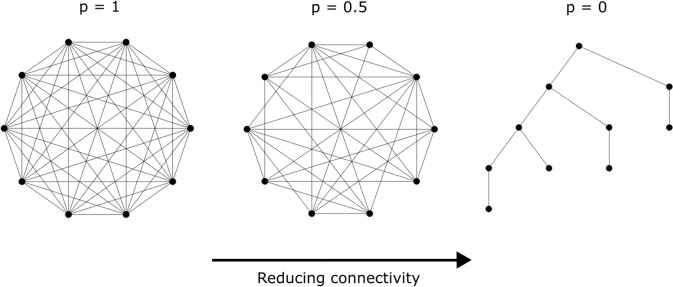
Connectivity reduction in small graphs. We start with a fully connected graph of size ${\boldsymbol{N}}$ and equal edge strength across the network ${{\boldsymbol{A}}}_{{\boldsymbol{ij}}{\boldsymbol{,}}{\boldsymbol{i}}{\boldsymbol{\ne }}{\boldsymbol{j}}}{\boldsymbol{=}}{\boldsymbol{1}}$. Picking each edge at random, the edge is removed with probability ${\boldsymbol{1}}{\boldsymbol{-}}{\boldsymbol{p}}$. If removing the edge makes the graph disconnected, the action is reversed. We repeat the process of selection for removal for ${\boldsymbol{N}}$ cycles.

Analysis of different networks from regular to random using the Watts-Strogatz^27^ or Barabási-Albert^28^ models would be the first step in assessing synchronizability. But the graph generation algorithms proposed in those methods are better suited for realistic large networks whereas our interest is in small graphs with finer control on randomness. (Results using these algorithms are presented later in the text). We propose an algorithm to generate graphs of varying connectivity (see Methods) where $p=1$ implies a fully connected graph (high mean-degree) and $p=0$ gives a sparsely connected graph (low mean-degree). Examples of graphs generated with 10 nodes and varying $P$ is shown in Fig. 2. The strength of connectivity between any node pair is equal.

**Figure 3:**
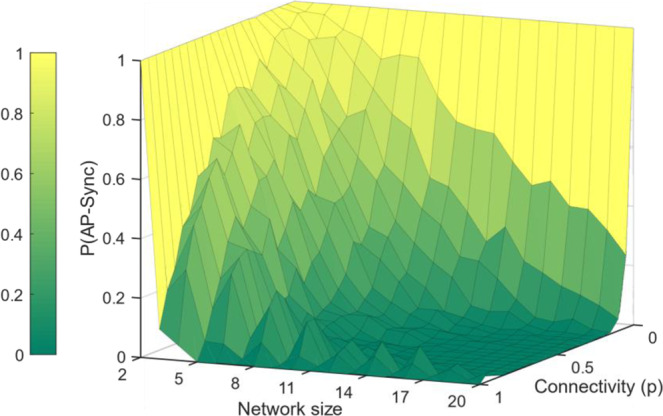
Effect of connectivity with network size on anti-phase synchronization. Probability of anti-phase synchronization reduces with network size and with degree of connectivity in random graphs. When connectivity is low, AP synchronization is stable for larger sizes. Differences in stability between odd and even sizes are evident. Fully connected even-sized networks are easier to synchronize as partitioning in to two groups is easier than fully connected odd-sized networks odd-sized networks. The odd-even effect reduces with decreasing connectivity.

For stronger connectivity, the AP synchronized solution quickly becomes unstable as shown in Fig. 3. The results reveal that regardless of connectivity, the probability of antiphase solution decreases to zero with increasing network size.

To confirm that the results hold for established random graph generation algorithms as well, we now look at networks generated using the Barabási-Albert algorithm and Watts-Strogatz algorithm.

#### Barabási-Albert model

**Figure 4:**
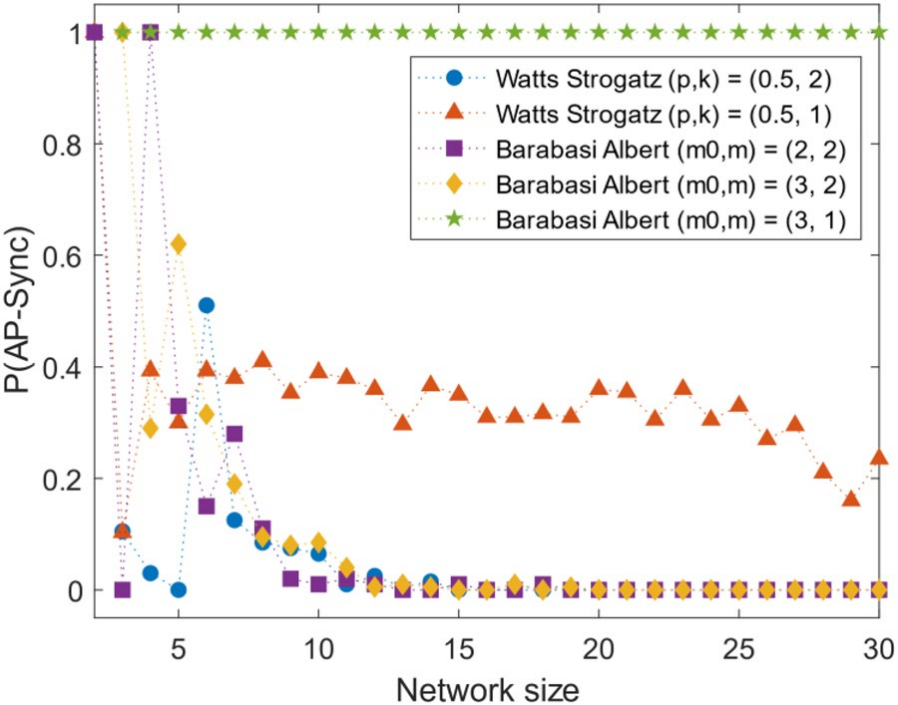
Results with alternative random-graph algorithms. Networks generated using the Barabási-Albert (BA) algorithm and the Watts-Strogatz (WS) algorithm are shown for different parameter values. The WS networks generated were generated from ${\boldsymbol{k}}={\boldsymbol{1}}$ and ${\boldsymbol{k}}={\boldsymbol{2}}$ connected nearest neighbors on each side initially. The plots are for networks in the small-world region of ${\boldsymbol{p}}={\boldsymbol{0}}{\boldsymbol{.}}\,{\boldsymbol{5}}$. The BA algorithm was used to generate networks starting from ${{\boldsymbol{m}}}_{{\boldsymbol{0}}}={\boldsymbol{2}}$ nodes and adding each node connected to ${\boldsymbol{m}}$ existing nodes. Typically, ${{\boldsymbol{m}}}_{{\boldsymbol{0}}}={\boldsymbol{m}}$, which is the m = 2 case shown here. It drops to zero very quickly. The ${\boldsymbol{m}}={\boldsymbol{1}}$ case remains very similar to the star topology as each new node only forms one link most probably to the same node. This allows for a higher probability of synchronization up to higher network sizes.

Many “real world” networks such as citation networks, the world wide web, and biological networks have hubs and have a ‘scale-free’ (power-law) nature. The Barabási-Albert^28^ algorithm generates graphs with growth and preferential attachment resulting in a scale free graph. Although the algorithm typically produces its power-law characteristics for large $N$, we apply it here to generate small network sizes. For a network of size $N$, the algorithm is seeded with a network of ${m}_{0}$ nodes and $N-{m}_{0}$ nodes are added sequentially where each node connects to $m$ existing nodes, with a preference to form links to nodes with a higher degree (rich-get-richer paradigm). For $m=1$, the B-A algorithm produces graphs close to the star topology, as the preferential attachment for the hub would mean that every new node has a high chance of forming a link to the hub. We set $m=2$ for more variation in the degree distribution, seeding the algorithm with ${m}_{0}=2,{\rm{A}}=[1,0{\rm{;}}0,1]$ and ${m}_{0}=3,{\rm{A}}=[0,1,0{\rm{;}}1,0,1{\rm{;}}0,1,0]$. The probability as shown in Fig. 4 agree with our main result.

#### Watts-Strogatz model

Watts and Strogatz showed that many real world networks have a ‘small-world’ nature^27^, where the networks display a low average path length while maintaining a high-clustering coefficient. The WS algorithm starts with a ring of nodes connected to $k$ nearest neighbors on each side and randomly rewires each edge with probability ${p}_{{WS}}$. Graphs generated using $k=1$ typically have long chains with few branches. This allows for better AP Sync. However, this would not be realistic. Setting  $k=2$ and simulating networks from ${p}_{{WS}}=0$ to ${p}_{{WS}}=1$, we find the same overall result. The results in the small-word regime at ${p}_{{WS}}=0.5$ are shown in Fig. 4. Both WS and BA model achieve AP synchronization for low connectivity, confirming our result. The real-world nature of these networks indicates that clustering into two groups may be present in real networks with repulsive interactions and low connectivity.

### Symmetry and distance effects

**Figure 5:**
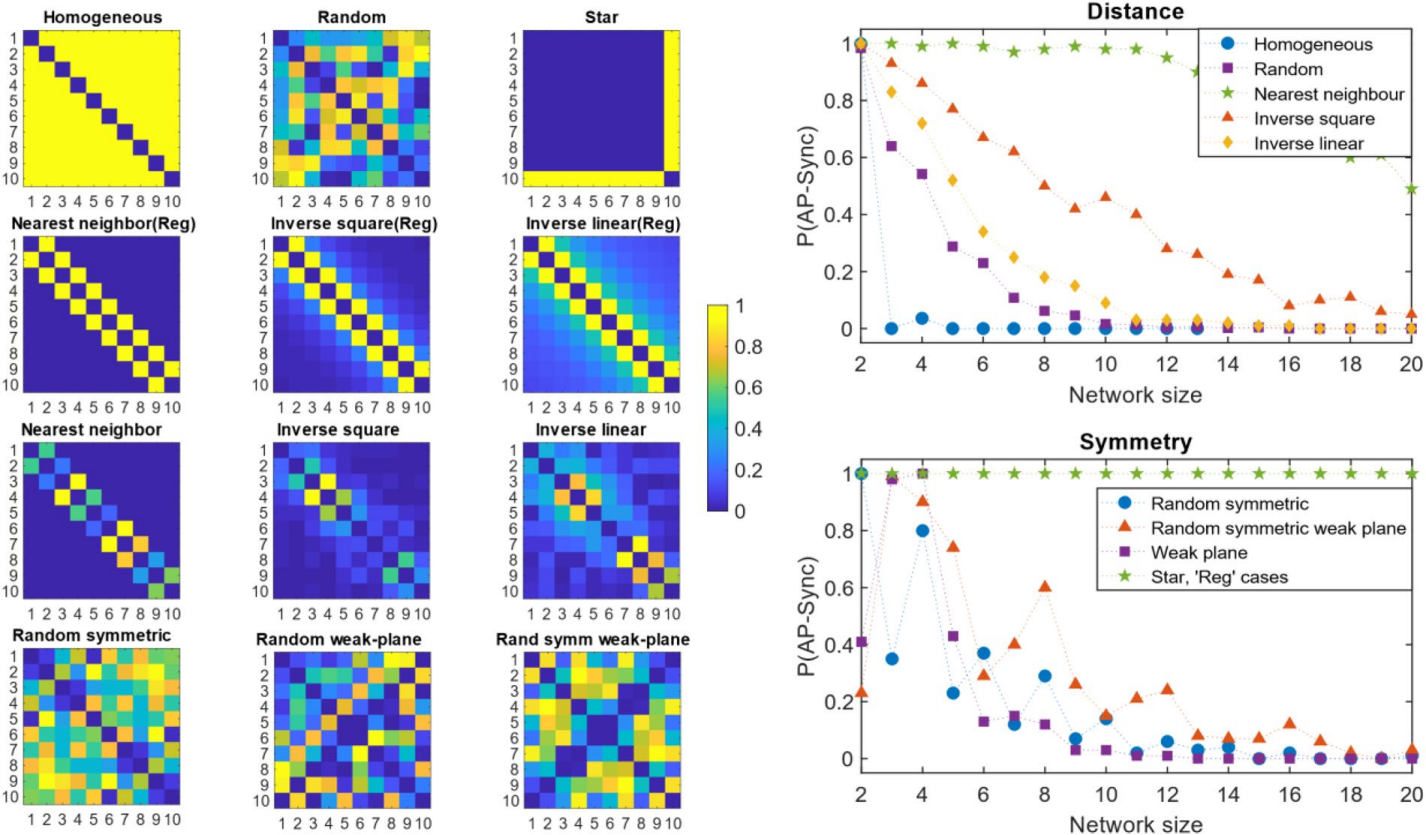
Effects of structure – symmetry and distance of coupling. The color matrices are plotted using the adjacency matrices of the networks, where the color represents the edge strength between nodes ${\rm{i}}$ and ${\rm{j}}$. Since the networks are undirected, all matrices are symmetric about the main diagonal. Symmetry in the network implies the adjacency matrix is bisymmetric (last row). The results show that symmetry enhances AP synchronization slightly. Coupling is varied as a function of distance for initially homogeneous (Reg) and random networks (see Methods). The star topology and regular cases give perfect AP synchronization regardless of network size. For random networks, nearest neighbor coupling allows the best chances of AP synchronization. As the interaction persists longer over distance, probability of AP synchronization reduces.

Although the general classical network models adopt random graphs, it has been shown that symmetry is ubiquitous in real world networks^29^ which may allow for AP synchronization. Homogeneous coupling is obviously symmetric to any degree, but networks with heterogeneous coupling can have degrees of symmetry as well. The simplest case is when the oscillator coupling strengths are mirrored about a plane, forming two symmetric groups. The coupling matrix $A$ is bisymmetric in this case. Allowing mirror symmetry enhances AP synchronization, P(AP-Sync) slightly for higher $N$ as shown in Fig. 5. Creating a plane of separation in the graph by setting the cross-diagonal elements of $A$ close to 0, has an impact on increasing AP synchronization. Along with symmetry, this enhances the ability of the network to AP synchronize as shown in Fig. 5. But regardless of these additional structural incentives, AP synchronization appears to vanish beyond approximately 20.

A clear demarcation into two structural groups would allow antiphase synchronization at large network sizes *given enough time*. A network with a star topology is a simple example where AP synchronization is possible for arbitrarily large network size (Fig. 5). But as expected, one of the clusters has only one node - the hub - and all other nodes are in the antiphase cluster.

Distance is another structural parameter that may influence the formation of opposing clusters. In real networks, interaction levels (measured by edge strength, ${A}_{{ij}}$) may decrease with distance. An inverse-square and inverse-linear dependence on distance was placed on fully connected graphs with homogenous and random edge strengths (see Methods). For the network resulting from the homogeneous case, AP synchronization is achieved at arbitrary sizes, given enough time, similar to the star-topology case. However, for the random case, our central result holds, the probability of AP synchronization vanishes above 20. Nearest-neighbour coupling allows AP synchronization to persist for slightly larger network sizes. (Fig. 5).

### Presence of both attractive and repulsive coupling

**Figure 6:**
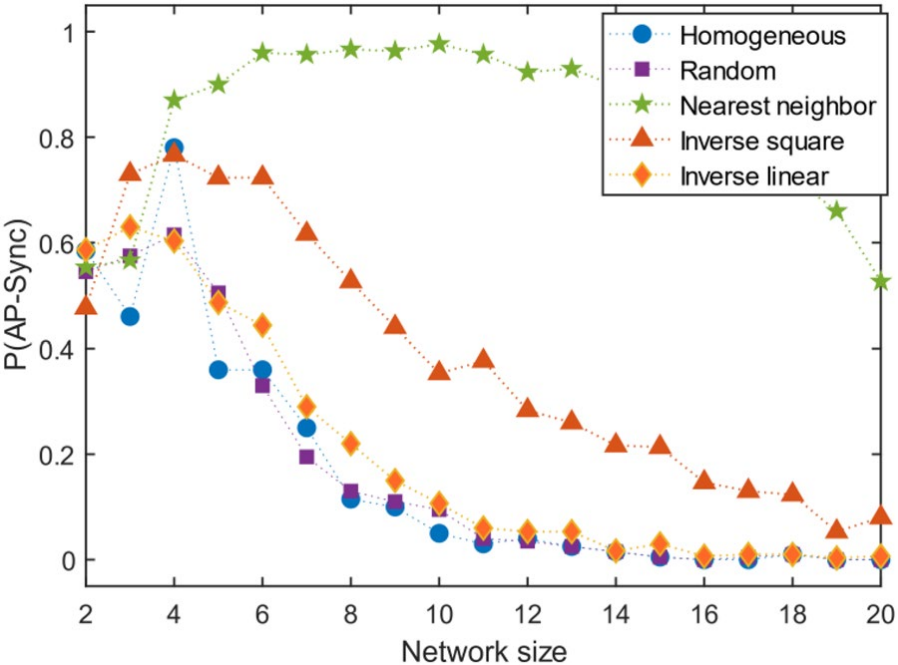
Simultaneous presence of attractive and repulsive coupling. For the more general case of arbitrary direction of coupling, the overall result presented holds, however a regime of preference for AP synchronization is observed.

Setting the range of ${A}_{i,j}$ to $[-\mathrm{1,1}]$ while $\phi =0$ allows incorporation of both attractive and repulsive coupling in the network. The presence of attractive coupling does not change the overall result; we find a non-monotonic curve as the network size increases for some cases (Fig. 6). I.e. there is a preference for antiphase synchronization over in-phase synchronization for moderate network sizes.

The addition of attractive coupling does not de-stabilize the system drastically for moderate network sizes. The upper bounds on network size remain effectively unchanged. This can be attributed to the tendency of attractive oscillators to join the corresponding antiphase clusters.

### Non-identical oscillators

**Figure 7:**
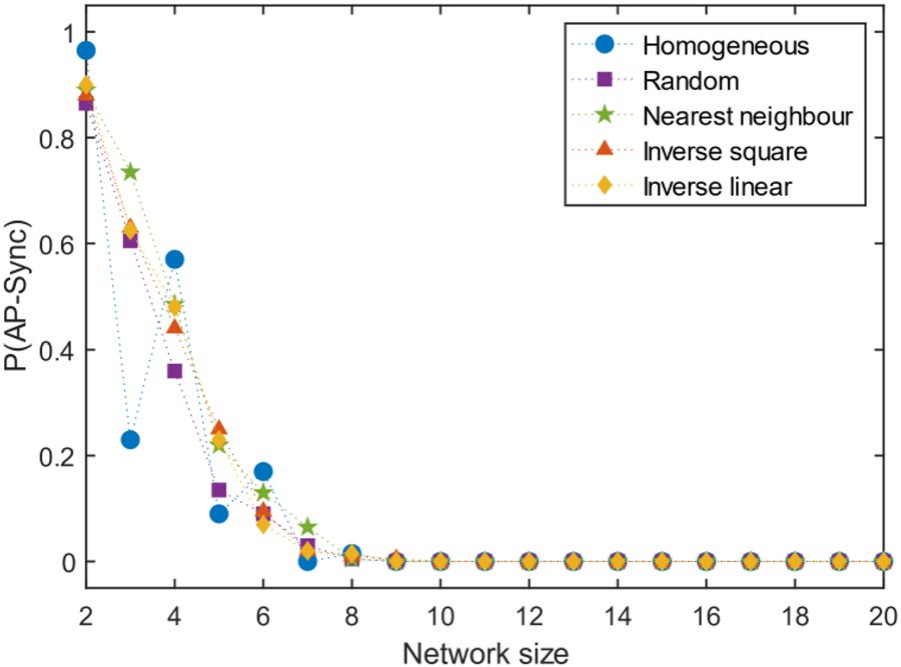
Effect of normally distributed frequency. Further generalization of the analysis to normally distributed frequencies of oscillators reduces the limit at which AP sync can be observed.

Allowing a normal distribution (σ = 0.05) in the natural frequency of the oscillators makes the size limits on the network more stringent. The probability of AP sync vanishes with lower network sizes than their corresponding identical-frequency case for all structural cases (Fig. 7).

Since most real-world systems would be composed of non-identical oscillators, this result implies that AP synchronization is rarely stable beyond very small network sizes in real systems. The inhomogeneity of the oscillators may be the decisive factor over the coupling topology for AP synchronization in real networks.

## Discussion

### Time required for antiphase synchronization

**Figure 8:**
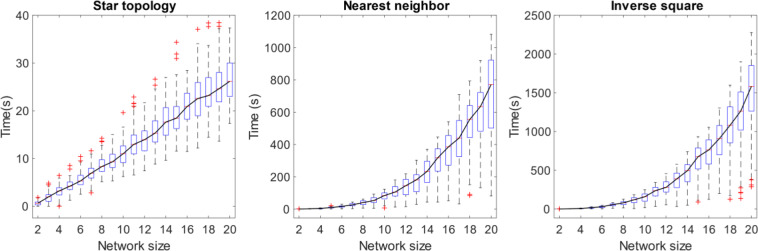
Increase in time to AP synchronization. The time taken to AP synchronize increases with network size for network topologies where antiphase synchronization is possible. The variance of the time increases as well as shown by the boxplot. The solid line connects the median time required. The box bounds the 75th percentile and the plus markers indicate the outliers.

For well-structured cases, such as the star topology we saw that AP sync happens regardless of network size. However, the time required increases with network size (Fig. 8)

**Figure 9:**
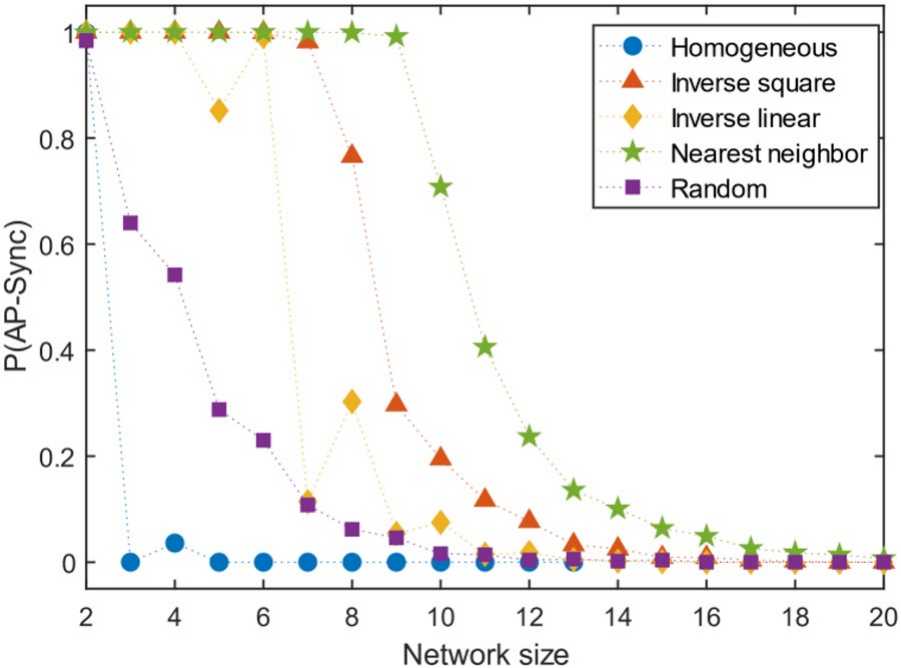
Fixed time ($t=100$) probability of AP synchronization. The fraction of the random initial conditions that can AP synchronize for regular graph networks falls with network size for fixed time. In real networks, this would allow other faster dynamical interactions (within the oscillator or with the environment, etc.), if any, to affect the formation of the AP synchronized steady state.

As a result of the increase in time required to synchronize, the probability of AP synchronization for *fixed time* shows a similar trend as our main result (Fig. 9). In a real network, the network would be composed of dynamical entities that have interactions at multiple time scales, within the network and with its environment. The increase in time to synchronize implies that even regular networks that could synchronize given enough time, become susceptible to dynamics at shorter time scales as the network increases in size.

### Link with graph-cutting algorithms

The AP synchronization problem is equivalent to an energy minimization problem. The oscillators once set up with their initial conditions attempt to find the minimum energy configuration. Similar to the Ising model or XY model from statistical mechanics, the configuration energy of the oscillators can be written as

2 $$H=-\sum _{i\ne j}{J}_{ij}\,\cos ({{\rm{\theta }}}_{j}-{{\rm{\theta }}}_{i})$$

Where $J$ is the configuration matrix, equivalent to the adjacency matrix in our case, ie. $J\equiv A$.

If we are only interested in solutions where ${{\rm{\theta }}}_{i}-{{\rm{\theta }}}_{j}$ takes two values, 0 or ${\rm{\pi }}$ at steady state, the energy function can be considered as involving only binary values. Let us suppose that we are interested in partitioning the network into two via a graph cut.

It has been proven that the partitioning problem is in general NP-Hard unless the energy function obeys some regularity conditions. If these conditions are satisfied, the problem can be solved using polynomial time algorithms for graph-cuts. See Kolmogorov-Zabih^30^ for proofs.

## Conclusion

This paper presents the first comprehensive analysis of the effects of network topology on AP synchronization of repulsively coupled oscillator networks. The effects of connectivity, symmetry, strength of interaction over distance, presence of attractive and repulsive coupling, and inhomogeneity of oscillators are considered. The analysis uses Stuart-Landau model for oscillators allowing general applicability to real networks with amplitude dynamics and weak-nonlinearity. The generalization from phase oscillators to Stuart-Landau oscillators allows an expansion of the regime of probable AP synchronization from 4 to 20+ with higher sizes possible under favourable network conditions. The favourable conditions were shown to be decreased connectivity of the network, decreasing strength of interaction over distance, and increased symmetry of the network structure. Inclusion of attractive coupling does not change the probability of AP synchronization radically but removes monotonicity along network size. Inclusion of inhomogeneity of the oscillator natural frequencies radically reduces the ceiling of network size for probable AP synchronization. For large networks, AP synchronization is unstable as observed in phase oscillator studies, with a few exceptions where the median connectivity is small, e.g. the star topology. Finally, the variation in timescales of AP synchronization under different coupling conditions and the links with computational complexity are discussed. Even in network sizes where AP synchronization is probable based on network conditions, the time to achieve synchronization grows exponentially, compounding their limited occurrence in real networks. The generality of the analysis may provide insight into cluster sizes in networks that have repulsive coupling, arising in physical, biological and socio-economic systems.

## Methods

### Connectivity reduction algorithm

From a fully connected graph, the median degree of a node is to be decreased from $N-1$ to 2 or as close to 2 as possible. We define a random trial with probability $p$ of selecting 1 vs 0, and it’s result is $P$. Each element of ${{\rm{A}}}_{{\rm{ij}}}$ is set to $P$ while checking if the graph becomes disconnected. If it does, reverse the action. If this procedure is followed in a sequential order from ${A}_{11}$ to ${A}_{{NN}}$, a star topology will always be formed at $p=0$. To allow for other random topologies, we pick elements from $A$ at random. We cycle through ${A}_{{ij}}$ $N$ times to ensure a sparse graph towards $p=0$.

### Influence of distance on edge strength

For the homogenous case, we have a fully connected graph, ${A}_{{ij},i\ne j}=1,{A}_{{ii}}=0$. For the random case, ${A}_{{ij}}$ is drawn from a uniform distribution in $[\mathrm{0,1}]$. For a linear graph, ${A}_{{ij}}/{|i-j|}^{2}$ gives inverse-square decrease and ${A}_{{ij}}/|i-j|$ gives inverse-linear decrease. Nearest-neighbour coupling implies, ${A}_{i,i\pm 1}=1$ and ${A}_{i,j}=0$ for all other terms.

### Simulation

The initial conditions for amplitude were drawn from a uniform distribution in $[\mathrm{0,1}]$ and for phase were drawn from a uniform distribution in $[\mathrm{0,2}{\rm{\pi }}]$. The solver used was the Runge-Kutta Dormand-Prince pair using ode45 in MATLAB. The number of iterations was at least 1000 for each set of initial conditions. The cluster was considered to be anti-phase synchronized if the phase difference between the pairs was within ${\rm{\pi }}\pm 0.2$. Time to AP synchronize a network of size $N$ increases nonlinearly with $N$. Simulation time was at least 2x the mean-time estimated for size $N$.

## Data Availability

The data used to generate the plots in this work are available upon request.

## Code availability

Computer code for reproducing the simulations is available upon request.
